# Supplementary material for: “Testing for malaria does not cure any pain” A qualitative study exploring low use of malaria rapid diagnostic tests at drug shops in rural Uganda
Source: PLOS Glob Public Health. 2022 Dec 13;2(12):e0001235. doi: 10.1371/journal.pgph.0001235 (PMC10021593; doi:10.1371/journal.pgph.0001235)
Supplement: S1 Appendix — (DOCX) [file pgph.0001235.s001.docx]

**S1 APPENDIX: IN-DEPTH INTERVIEW GUIDES**

**INTERVIEW GUIDE FOR DRUG SHOP VENDORS**

*I. Warm-up*

1. To start, can you tell me a little about your experience working as a drug shop vendor?

- How long have you worked at this drug shop?
- What illnesses do you most commonly treat?

*II. History of RDT Use at Drug Shop*

2. I would like to talk more specifically about malaria testing and treatment at this drug shop. Can you tell me about the reasons your drug shop began using malaria RDTs?

- How long has your drug shop been using RDTs?

3. How did you learn how to use malaria RDTs?

- Have you received training on the use of RDTs?
- Have you received training on malaria treatment or diagnosis more generally?

*III. Vendor Perceptions of RDTs*

4. How comfortable do you feel using RDTs to diagnose malaria among your clients?

- What’s easy about using RDTs? Is there anything that’s difficult about using RDTs?
- [If using RDTs is difficult] What additional support do you need to use RDTs?

5. What are your views about the use of RDTs to test for malaria at your drug shop?

- What do you like about using RDTs? Is there anything you don’t like about using RDTs?
- Do you think malaria RDTs are necessary?

6. Do you think RDTs accurately diagnose malaria?

- How does the accuracy of RDTs compare to diagnosis based on symptoms alone?

*IV. Describing RDTs to Drug Shop Clients*

7. I would like to talk more about how you treat your clients for malaria. Can you describe for me what happens when clients come to your drug shop with a fever?

- Do clients describe their symptoms to you?
- What do clients say is the cause of their fever?
- [If malaria] How do clients know if a fever is caused by malaria or something else?

8. How often do you discuss RDTs with your clients?

- How would you describe the purpose of an RDT to a client?
- Do you encourage your clients to get an RDT?
- Which clients would you encourage to get an RDT?

9. How often do clients who come to your drug shop with a fever get an RDT?

- Does this vary depending on the season?
- Are certain types of people more likely to get an RDT?

*V. Non-Use of RDTs*

10. What do you think are the reasons some clients don’t get an RDT?

- Are there different reasons for men and women? Children and adults?

11. Do you think that clients buying antimalarials without an RDT is a problem? Why or why not?

1. What do you think would encourage more people to get RDTs at drug shops?

*VI. Use of RDTs*

13. I would like to talk more about the clients who get RDTs from your shop. What do you think are the reasons some clients get RDTs?

- Are there different reasons for men and women? Children and adults?

14. If a client gets an RDT and tests positive for malaria, how do they react?

- How would you describe the results to them?
- Do they believe that they have malaria if the test is positive?
- What medications would you recommend? Any other medications?
- What is your understanding of what this medicine is used to treat?

15. If a client buys an RDT and tests negative for malaria, how do they react?

- How would you describe the results to them?
- Do they believe that they do not have malaria if the test is negative?
- What medications would you recommend? Any other medications?
- What is your understanding of what this medicine is used to treat?

16. Are there times when a client tests negative but you still believe they have malaria? Can you describe these situations for me?

- How would you respond in this situation?

*VII. Antimalarial Purchases After Negative Results*

17. How often do clients who test negative for malaria buy antimalarials?

- How do you respond to these clients?
- Does this vary depending on the season?
- Are certain types of people more likely to buy antimalarials after a negative test result?

18. What do you think are the reasons clients buy antimalarials after they test negative?

- Are there different reasons for men and women? Children and adults?

19. Do you think that clients buying antimalarials after testing negative is a problem? Why or why not?

20. What do you think would encourage more people to only buy antimalarials if they test positive?

*VIII. Perceptions of Intervention Options*

In other places, governments and non-profit organizations have tried different programs to increase the number of drug shop clients who buy RDTs from drug shops, and decrease the number of drug shop clients who buy antimalarials after they test negative. I’m going to briefly tell you about these programs, and I would like to hear what you think about them.

21. What do you think about community sensitization to promote the importance of malaria diagnosis and RDTs to the community?

- What do you like about this program? Is there anything you don’t like about this program?
- What messages do you think would be important to include?
- What are the best ways to reach people with health information?
- Do you think this program would work?
- What questions or concerns do you have as a drug shop vendor about this program?

22. What do you think about training for drug shop vendors? The topics of trainings could include (1) malaria diagnosis and treatment (2) treatment and referral for fevers not caused by malaria, and (3) improving communication and negotiation with drug shop clients.

- What do you like about this program? Is there anything you don’t like about this program?
- Are there topics that you think are particularly important?
- Do you think this program would work?
- What questions or concerns do you have as a drug shop vendor about this program?

23. What do you think about a program through the government or non-profit organizations that would provide free or subsidized RDTs to be distributed at drug shops?

- What do you like about this program? Is there anything you don’t like about this program?
- Do you think this program would work?
- What questions or concerns do you have as a drug shop vendor about this program?

24. What do you think about a referral program to help link drug shop clients who test negative for malaria to public health facilities for treatment?

- What do you like about this program? Is there anything you don’t like about this program?
- Do you think this program would work?
- What questions or concerns do you have as a drug shop vendor about this program?

25. What do you think about health facility improvements to encourage more community members to visit health facilities? For example, reducing waiting time and medication stockouts.

- What do you like about this program? Is there anything you don’t like about this program?
- Do you think this program would work?
- What questions or concerns do you have as a drug shop vendor about this program?

26. Do you have suggestions for other programs to increase the number of drug shop clients who buy RDTs, and decrease the number of drug shop clients who buy antimalarials after they test negative?

*Conclusion*

Those are all the questions that I have. Thank you very much for taking the time to talk to me today. Before we end, do you have any questions for me? Is there anything else you think is important for me to know?

**INTERVIEW GUIDE FOR VILLAGE HEALTH TEAM MEMBERS**

*I. Warm-up*

1. To start, can you tell me a little about your experience working as a VHT?

- How long have you been a VHT?
- What illnesses do you most commonly see in your community?
- Why did you decide to become a VHT?

*II. VHT Perceptions of RDTs*

2. What are your views about the use of RDTs to test for malaria in children in your community?

- What do you like about using RDTs? What do you dislike about using RDTs?
- Do you think malaria RDTs are necessary?

3. Do you think RDTs accurately diagnose malaria?

- How does using RDTs to diagnose malaria compare to diagnosis based on symptoms alone?

*III. Malaria Diagnosis and Treatment among Children by VHTs*

4. I would like to talk more about your role treating children in your community for malaria. How do parents react if their child tests positive for malaria?

- How would you describe the results to them?
- Do parents trust that their child has malaria if the test is positive?

5. How do parents react if their child tests negative for malaria?

- How would you describe the results to them?
- Do parents trust that their child does not have malaria if the test is negative?

6. Are there times when a child tests negative but you still believe they have malaria? Can you describe these situations for me?

- How would you respond in this situation?

*IV. Perceptions of Malaria Diagnosis and Treatment in the Community*

While VHTs treat children under five for malaria, some people go to drug shops or health facilities when they are sick. I want to talk more generally about the views on malaria and RDTs in this community.

7. What do people in your community think about malaria?

- How bad is malaria for the people in your community?

8. How do people in your community know when they have malaria?

- How do they know if a fever is caused by malaria or something else?
- What symptoms are specific to malaria?
- Does confidence that a fever is caused by malaria vary depending on the season?

9. How do people in the community feel about the use of RDTs to test for malaria?

- What do they like about using RDTs? What do they dislike about using RDTs?
- Do they think malaria RDTs are necessary?

*V. Non-Use of RDTs at Drug Shops*

10. What do you think are the reasons people will buy antimalarials at drug shops without an RDT?

- Are there different reasons for men and women? Children and adults?

11. Do you think that people buying antimalarials at drug shops without an RDT is a problem? Why or why not?

12. What do you think would encourage more people to buy RDTs at drug shops?

*VI. Antimalarial Purchases After Negative Results*

13. Some people who test negative still buy antimalarials at the drug shop. What do you think are the reasons people buy antimalarials at drug shops after they test negative?

- Are there different reasons for men and women? Children and adults?

14. Do you think that people buying antimalarials after testing negative is a problem? Why or why not?

15. What do you think would encourage more people to only buy antimalarials if they test positive?

*VII. Perceptions of Intervention Options*

In other places, governments and non-profit organizations have tried different programs to increase the number of drug shop clients who buy RDTs from drug shops, and decrease the number of drug shop clients who buy antimalarials after they test negative. I’m going to briefly tell you about these programs, and I would like to hear what you think about them.

16. What do you think about community sensitization to promote the importance of malaria diagnosis and RDTs to the community?

- What do you like about this program? What do you dislike about this program?
- What messages do you think would be important to include?
- What are the best ways to reach people with health information?
- Do you think this program would work?
- What questions or concerns do you have about this program?

17. What do you think about training for drug shop vendors? The topics of trainings could include (1) malaria diagnosis and treatment (2) treatment and referral for fevers not caused by malaria, and (3) improving communication and negotiation with drug shop clients.

- What do you like about this program? What do you dislike about this program?
- Are there topics that you think are particularly important?
- Do you think this program would work?
- What questions or concerns do you have about this program?

18. What do you think about a program through the government or non-profit organizations that would provide free or subsidized RDTs to be distributed at drug shops?

- What do you like about this program? What do you dislike about this program?
- Do you think this program would work?
- What questions or concerns do you have about this program?

19. What do you think about a referral program to help link drug shop clients who test negative for malaria to public health facilities for treatment?

- What do you like about this program? What do you dislike about this program?
- Do you think this program would work?
- What questions or concerns do you have about this program?

20. What do you think about health facility improvements to encourage more community members to visit health facilities? For example, reducing waiting times and medication stockouts.

- What do you like about this program? What do you dislike about this program?
- Do you think this program would work?
- What questions or concerns do you have about this program?

21. Do you have suggestions for other programs to increase the number of drug shop clients who buy RDTs from drug shops, and decrease the number of drug shop clients who buy antimalarials after they test negative?

*Conclusion*

Those are all the questions that I have. Thank you very much for taking the time to talk to me today. Before we end, do you have any questions for me? Is there anything else you think is important for me to know?

**INTERVIEW GUIDE FOR DRUG SHOP CLIENTS**

*I. Warm-up*

1. To start, can you tell me what you think about malaria?

- How bad is malaria for the people in your community?
- What can people do to prevent malaria? Is this hard to do?

*II. Illness History*

2. You recently went to a drug shop to buy medicine because you/your child had a fever. I would like to hear the story of how you ended up at the drug shop that day, beginning with when the fever first started. Can you tell me about when you/your child started to feel sick?

- How did you/they feel?
- What symptoms did you/they have?

3. What did you think was the cause of the fever?

- Have you/they ever felt this way before?
- [If malaria] How do you know if a fever is caused by malaria or something else?

4. Before going to the drug shop, did you go for treatment anywhere else? If so, can you describe for me what happened when you went there?

- [If yes] Did you receive an RDT? Did you receive any medications?
- [if yes] Were you satisfied with the outcome?
- [If yes] Why did you choose to go there for treatment?
- Did you take any medicine at home?
- Can you tell me about any discussions you had about this decision?

5. What made you decide to go to the drug shop?

- Can you tell me about any discussions you had about this decision?
- How did you choose this drug shop over other drug shops nearby? Over a facility?
- How much time passed from when you/your child became sick to when you went to the drug shop?

*III. Interactions with the Drug Shop Vendor*

6. At the drug shop, can you describe for me, in as much detail as you can remember, your conversation with the drug shop vendor?

- Did you discuss your/your child’s symptoms with the vendor? What did you say?
- Did the drug shop vendor ask you any questions about how you/your child were feeling?

7. Did you and the drug shop vendor talk about taking a malaria RDT?

- [If yes] What did you and the drug shop vendor discuss?

*IV. RDT and Medication Purchases at the Drug Shop*

8. Did you get an RDT at the drug shop? What are the reasons you had/didn’t have an RDT?

- Can you tell me about any discussions you had about this decision?
- [If money is the reason they didn’t get an RDT] If you had the money for the RDT, would you prefer to get tested or use the money in another way for your family?

9. [For individuals tested at the drug shop]. Can you describe for me how the drug shop vendor gave you the RDT and told you about the results?

- How did you react when you found out the test was negative/positive?
- Were the results what you were expecting?
- Do you think that the results from the RDT were correct?

10. What medicines did you buy from the drug shop? *(Note to interviewer: we are interested in all medicines, including antimalarials, antibiotics, and painkillers.)*

- How did you decide which medicine(s) to buy?
- Did the vendor recommend this medicine or did you request for it?
- What is your understanding of what this medicine is used to treat?

*V. Past Experiences with RDTs*

11. Before this visit to the drug shop, what has been your experience with malaria RDTs?

- Where have you received malaria RDTs?
- What were some of the reasons you received malaria RDTs in the past?

12. Have you ever received a positive RDT result? Can you describe the experience for me?

- [If yes] How did you feel when you tested positive?
- [If yes] Were the results what you were expecting?
- [If yes] Did you believe that you had malaria when the test was positive?

13. Have you ever received a negative RDT result? Can you describe the experience for me?

- [If yes] How did you feel when you tested negative?
- [If yes] Were the results what you were expecting?
- [If yes] Did you believe that you did not have malaria when the test was negative?

14. Have you ever had an experience where you or a family member believed you had malaria, only to find out that it was something else? If so, can you tell me about that experience?

- [If yes] What illness did you have? How long did it take to correctly determine the cause?
- [If yes] How has that experience changed your feelings about malaria testing?

*VI. Perceptions of RDTs*

15. In general, how do you feel about the use of RDTs to test for malaria?

- What do you like about malaria RDTs? Is there anything you don’t like about malaria RDTs?
- Do you think malaria RDTs are necessary?
- [If participant purchased an RDT at the shop] Are there times you don’t get an RDT at the drug shop?

16. What do you think would encourage more people to use RDTs at drug shops?

*VII. Trusted Sources of Health Information*

17. In your community, who do you trust with health information?

*Conclusion*

Those are all the questions I have for you. Thank you very much for taking the time to talk to me today. Before we end, do you have any questions for me? Is there anything else you think is important for me to know?

**INTERVIEW GUIDE FOR HEALTH AND COMMUNITY OFFICIALS**

*I. Warm-up and Overview of Position*

1. How would you describe your role in improving the health of the people in Bugoye?

2. What do you think are some of the biggest health challenges here (in Bugoye, western Uganda, or Uganda depending on the official and the organization)?

3. How much of a priority is improving malaria case management practices at private sector drug shops to your organization?

4. What do you think of the current regulation of private-sector drug shops?

*II. Review of Results*

5. What stands out to you about these results on malaria diagnostic practices at drug shops?

- Are these results what you expected?
- How do these results compare to what you have seen elsewhere?

6. Do you have any questions for me about these results?

*III. Perceptions of Best Ways to Intervene*

7. Do you think that people buying malaria medications at drug shops without being tested using an RDT is a problem? Why or why not?

8. How do you think we could encourage more people to buy RDTs at drug shops?

9. Do you think that people buying malaria medications at drug shops after testing negative is a problem? Why or why not?

10. How do you think we could encourage more people to only buy malaria medications at drug shops if they test positive?

IV*. Perceptions of Intervention Options*

In other places, governments and non-profit organizations have tried different programs to increase the number of drug shop clients who buy RDTs from drug shops, and decrease the number of drug shop clients who buy malaria medications after they test negative. I’m going to briefly tell you about these programs, and I would like to hear what you think about them, and whether they might work here.

11. What do you think about a messaging campaign to promote the importance of malaria diagnosis to the community?

- What do you like about this program? What do you dislike about this program?
- What messages do you think would be important to include?
- Do you think this program would be feasible?
- Do you think this program would work?
- What questions or concerns do you have about this program?

12. What do you think about training for drug shop vendors? The topics of trainings could include (1) malaria diagnosis and treatment (2) treatment and referral for non-malarial fevers, and (3) improving communication and negotiation with drug shop clients.

- What do you like about this program? What do you dislike about this program?
- Are there topics that you think are particularly important?
- Do you think this program would be feasible?
- Do you think this program would work?
- What questions or concerns do you have about this program?

13. What do you think about a program through the government or non-profit organizations that would provide free or subsidized RDTs to be distributed at drug shops?

- What do you like about this program? What do you dislike about this program?
- Do you think this program would be feasible?
- Do you think this program would work?
- What questions or concerns do you have about this program?

14. What do you think about a referral program to help link drug shop clients who test negative for malaria to public health facilities for treatment?

- What do you like about this program? What do you dislike about this program?
- Do you think this program would be feasible?
- Do you think this program would work?
- What questions or concerns do you have about this program?

15. What do you think about health facility improvements to reduce wait times and medication stockouts to encourage more community members to visit health facilities?

- What do you like about this program? What do you dislike about this program?
- Do you think this program would be feasible?
- Do you think this program would work?
- What questions or concerns do you have about this program?

16. Do you have suggestions for other programs to increase the number of drug shop clients who buy RDTs from drug shops, and decrease the number of drug shop clients who buy malaria medications after they test negative?

*Conclusion*

Those are all the questions that I have for you. Thank you very much for taking the time to talk to me today. Before we end, do you have any questions for me? Is there anything else you think is important for me to know?
