# Supplementary material for: “Testing for malaria does not cure any pain” A qualitative study exploring low use of malaria rapid diagnostic tests at drug shops in rural Uganda
Source: PLOS Glob Public Health. 2022 Dec 13;2(12):e0001235. doi: 10.1371/journal.pgph.0001235 (PMC10021593; doi:10.1371/journal.pgph.0001235)
Supplement: S2 Appendix — (DOCX) [file pgph.0001235.s002.docx]

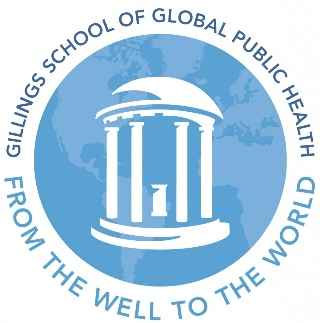
**Malaria Testing and Treatment at Drug**

**Shops in Bugoye**

A study from the MUST-UNC Research Collaboration

**Methods**

**46 drug shops** in Bugoye sub-county that sell antimalarials collected data and blood samples from clients for 2 weeks.

Data collected:

● Presence of sick individual

● Client age, sex, and village

● Days of illness

● Symptoms

● RDT results

● Medication purchases

Blood samples were collected if the sick individual was present at the drug shop and did not have an RDT. Samples were tested for malaria at Bugoye Health Centre III.

Data collection groups

- Group 1 - 12 drug shops near Bugoye and Mulehe collected data from July 12 to 25
- Group 2 - 15 drug shops near Ibanda collected data from August 3 to 16
- Group 3 - 10 drug shops near Katooke collected data from August 24 to September 6
- Group 4 - 9 drug shops near Kisamba and Muhambo collected data from September 9 to 22

**Results**

Client characteristics:

● 48% male, 52% female

● Average age: 23.5 years

● Average time sick: 3.4 days

● 11% under 5 years

● 4% pregnant

● 71% of people buying medications for themselves

**Key Findings**

1. **Low use of RDTs at drug shops**

● 26% of clients received an RDT at the drug shop + 20% reported receiving an RDT from a VHT or health centre

1. **High antimalarial sales, even to clients who tested negative or did not test**

● 36% of clients who tested negative for malaria still purchased antimalarials

● Most clients who did not get an RDT purchased antimalarials (87%)

1. **Many clients who purchased antimalarials without an RDT did not have malaria**

● 65% of clients treated presumptively for malaria tested negative based on collected blood samples (n=221)
