# Supplementary material for: “Testing for malaria does not cure any pain” A qualitative study exploring low use of malaria rapid diagnostic tests at drug shops in rural Uganda
Source: PLOS Glob Public Health. 2022 Dec 13;2(12):e0001235. doi: 10.1371/journal.pgph.0001235 (PMC10021593; doi:10.1371/journal.pgph.0001235)
Supplement: S3 Appendix — (DOCX) [file pgph.0001235.s003.docx]

**S3 APPENDIX: INTERVIEWER SUMMARY AND DEBRIEF FORM**

*Date: _______________ Participant ID: _______________ Interviewer: _______________*

*Part I. Thematic Summary*

1. Provide an overall summary of the “story” of the interview, including the key themes that emerged during the discussion:

*Part II. Debrief Questions*

1. Was this a good interview? Why or why not?
2. Were there any problems with this interview that the analysis should take into account? Did any of the questions not work well?
3. Was there anything new you hadn't encountered before? (e.g. themes)
4. Are there changes to the interview guide or procedures that should be made for future interviews?
5. Any additional notes?
