# Supplementary material for: “Testing for malaria does not cure any pain” A qualitative study exploring low use of malaria rapid diagnostic tests at drug shops in rural Uganda
Source: PLOS Glob Public Health. 2022 Dec 13;2(12):e0001235. doi: 10.1371/journal.pgph.0001235 (PMC10021593; doi:10.1371/journal.pgph.0001235)
Supplement: S4 Appendix — (DOCX) [file pgph.0001235.s004.docx]

**S4 APPENDIX: CODEBOOK**

| Qualitative codes\subcodes |
| --- |
| Malaria perceptions |
| Malaria perceptions\symptoms of malaria |
| Malaria perceptions\illnesses confused with malaria |
| Malaria perceptions\severity of malaria |
| Malaria perceptions\prevalence of malaria |
| Malaria perceptions\causes of malaria |
| Malaria perceptions\malaria and seasonality |
| Malaria perceptions\severity of malaria |
| Malaria prevention |
| Decision to go the drug shop |
| Decision to go the drug shop\preference over health facility |
| Decision to go the drug shop\shop selection |
| Decision to go the drug shop\discussion with others |
| Decision to go the drug shop\length of time |
| Perceptions of drug shops |
| Perceptions of drug shops\role of drug shops |
| Perceptions of drug shops\reasons people prefer drug shops |
| Challenges with drug shops |
| Challenges with drug shops\no RDTs |
| Challenges with drug shops\enforcement and licensing |
| Challenges with drug shops\unqualified vendors |
| Challenges with drug shops\expired medications |
| Challenges with drug shops\improper storage |
| RDT perceptions |
| RDT perceptions\positive |
| RDT perceptions\negative |
| RDT perceptions\necessary |
| RDT perceptions\accurate |
| Reasons vendors began selling RDTs |
| Training on the use of RDTs |
| Comfort using RDTs |
| Challenges using RDTs |
| Trust in RDTs |
| Client and vendor interactions |
| Client and vendor interactions\symptoms |
| Client and vendor interactions\RDT discussion |
| Referrals |
| RDT use |
| RDT use\Reasons for getting an RDT |
| RDT use\Reasons for NOT getting an RDT |
| Positive RDT at shop |
| Positive RDT at shop\vendor explanation |
| Positive RDT at shop\reaction |
| Positive RDT at shop\medication |
| Negative RDT at shop |
| Negative RDT at shop\vendor explanation |
| Negative RDT at shop\reaction |
| Negative RDT at shop\medication |
| No RDT |
| No RDT\medication |
| Use of RDTs at drug shops |
| Use of RDTs at drug shops\frequency |
| Use of RDTs at drug shops\seasons |
| Use of RDTs at drug shops\groups |
| Use of RDTs at drug shops\reasons |
| Antimalarials without an RDT |
| Antimalarials without an RDT\groups |
| Antimalarials without an RDT\reasons |
| Antimalarials without an RDT\problematic |
| Antimalarials without an RDT\suggestions |
| Antimalarials without an RDT\vendor reactions |
| Antimalarials after a negative result |
| Antimalarials after a negative result\groups |
| Antimalarials after a negative result\reasons |
| Antimalarials after a negative result\problematic |
| Antimalarials after a negative result\suggestions |
| Antimalarials after a negative result\frequency |
| Antimalarials after a negative result\vendor reactions |
| Antimalarials after a negative result\seasonality |
| Program 1: Community sensitization |
| Program 1: Community sensitization\suggested spontaneously |
| Program 1: Community sensitization\positive |
| Program 1: Community sensitization\challenges |
| Program 1: Community sensitization\messages |
| Program 1: Community sensitization\ways to reach people |
| Program 1: Community sensitization\effective |
| Program 2: Vendor training |
| Program 2: Vendor training\suggested spontaneously |
| Program 2: Vendor training\positive |
| Program 2: Vendor training\challenges |
| Program 2: Vendor training\topics |
| Program 2: Vendor training\effective |
| Program 3: Free RDTs |
| Program 3: Free RDTs\suggested spontaneously |
| Program 3: Free RDTs\positive |
| Program 3: Free RDTs\challenges |
| Program 3: Free RDTs\effective |
| Program 4: Referrals |
| Program 4: Referrals\positive |
| Program 4: Referrals\challenges |
| Program 4: Referrals\effective |
| Program 5: Health center improvements |
| Program 5: Health center improvements\positive |
| Program 5: Health center improvements\challenges |
| Program 5: Health center improvements\effective |
| Other program suggestions |
| Reactions to Aim 1 results |
| Reactions to Aim 1 results\most concerning |
| Reactions to Aim 1 results\surprising or not |
| VHT or vendor experience |
| Health or community official role |
| Perceptions on licensing and inspection of drug shops |
| Health challenges in the community |
| Previous experience with RDTs |
| Previous experience with RDTs\Reasons for getting an RDT in the past |
| Previous experience with RDTs\Reasons for NOT getting an RDT in the past |
| Previous positive RDTs |
| Previous negative RDTs |
| Final comments |
| Good quotes |
