# Supplementary material for: “Testing for malaria does not cure any pain” A qualitative study exploring low use of malaria rapid diagnostic tests at drug shops in rural Uganda
Source: PLOS Glob Public Health. 2022 Dec 13;2(12):e0001235. doi: 10.1371/journal.pgph.0001235 (PMC10021593; doi:10.1371/journal.pgph.0001235)
Supplement: Attachment — Submitted filename: Response to Reviewers.docx [file pgph.0001235.s005.docx]

October 7, 2022

Dear PLOS Global Public Health Editors,

Thank you for the opportunity to submit revisions for our article *“Testing for malaria does not cure any pain” A qualitative study exploring low use of malaria rapid diagnostic tests at drug shops in rural Uganda.* A summary of the manuscript revisions made in response to the reviewer comments is included below. We believe the article has been strengthened because of the thoughtful comments of the reviewers. Please let me know if you have any further questions.

Sincerely,

Victoria Shelus

| **Manuscript Section** | **Reviewer Comments** | **Author Response and Manuscript Revisions** |
| --- | --- | --- |
| General | In the online submission form, you indicated that "The data presented in this study are available on request from the corresponding author. Qualitative transcripts are not publicly available due to the consent provided by participants on the use of confidential data.". All PLOS journals now require all data underlying the findings described in their manuscript to be freely available to other researchers, either 1. In a public repository, 2. Within the manuscript itself, or 3. Uploaded as supplementary information. | We have reached out to the editors separately about an exemption to the public data requirement because public deposition would breach compliance with the protocol approved by the research ethics board. The data availability statement has been revised to indicate that data is only available within the submission during peer review, and to provide an IRB contact should interested researchers wish to apply for data access after publication. |
| General | We have noticed that you have uploaded Supporting Information files, but you have not included a list of legends. Please add a full list of legends for your Supporting Information files after the references list. | A list of file names and titles for supporting information have been added. None of the supporting information files are figures or tables that require legends (listed as optional in submission guidelines). |
| General | Grammatical errors throughout the manuscript, both in the abstract and the main text. Needs revision. Refer to lines- L36 (Suggestion: Replace ‘fevers presented’ with ‘fever cases presenting), L195-196 (consider revising), L206 (check the tenses), L544 – 545 (incomplete sentence) | The entire paper has been reviewed for grammatical errors and revisions to specific lines cited have been made. |
| Introduction | Page number 3, Line number 62, please mention the exact incidence number reported in 2019-20. crosscheck with the WHO data. | Exact incidence numbers have been revised to reflect those included in the WHO World Malaria Report 2021. |
| Introduction | Page number 3, Line number 68, site of incidence Is not clear in the statement; please mention specific sites like Uganda. | This sentence has been revised to specify Uganda as the site. |
| Introduction | Line Number 82. Authors have highlighted that "RDTs quickly diagnose malaria in settings where high-quality laboratory services are not routinely available and have significantly reduced antimalarial over-prescriptions" later statement needs to be checked as its not conclusive as appeared in the cited studies. | This statement has been revised to more closely reflect the conclusions of the papers cited. |
| Methods | The study did not mention participants' recruitment details and study designs. Please add some details about it. | Detail about the study design (cross-sectional qualitative) and about how participants were recruited was added. The study team called potential participants to briefly describe the study, confirm interest and eligibility, and schedule an interview. |
| Methods | it seems the study is part of a project, and details are missing. Please name the details and related study you mentioned on page number, line number 126. Please specify, that there is no overlapping of the reported data.  Line number 125, Please specify the related study site details. | Additional detail has been added to clarify that the related study was conducted at the same site, employed quantitative methods, and informed the sampling strategy and qualitative themes for the current study. A citation has been added for the dissertation that describes the study in more detail. |
| Methods | Sample & Recruitment: L139 mentions ‘recent clients’; was any cut off time period taken into consideration or any operational definition developed for the same?  Page number 5, Line number 139, The term "recent clients is not clear; please elaborate. | This sentence has been revised to specify that clients were eligible to participate in the study if they had visited a drug shop with suspected malaria in the previous two weeks. |
| Methods | In page number 6, line number 147-148. If 96% of vendors are unlicensed, how do they maintain their clients' data records? Please mention how they maintain the client data. | Additional detail has been added to clarify that vendors received a paper form to record the names and contact information of clients for study recruitment. |
| Methods | Table 1: L164 – L167: While calculating the cut points for RDT sales, what was the denominator used in the previous studies? Does the word ‘clients’ include only the fever cases visiting the drug shops or all clients visiting the drug shops? This needs to be specified otherwise it may give erroneous information on the uptake of RTDs. | Additional detail has been added to clarify that clients were those with suspected malaria because they either reported fever or requested antimalarials. |
| Methods | Study tools: L169 mentions that ‘discussion guides’ were used for the study; however, discussion guides are used for FGDs. More appropriate tool for the current study would have been interview schedule / interview guide as the method for data collection used in the study has been mentioned as in-depth interviews. Consider replacing the word ‘discussion guides’ with ‘interview schedule / interview guides’. | “Discussion guides” have been changed to “In-depth interview guides” in the manuscript and supplement. |
| Methods | Please elaborate on how the sample size was calculated. | The sample size was calculated based on qualitative methods research that found saturation occurs between six and twelve interviews within a homogenous sample. Therefore, we aimed to interview six individuals from each strata. Additional detail has been added to the methods section to clarify this. |
| Methods | it seems that the clustering of the participants was planned initially and then recruited participants. In clustering, the geographic diversity data is missing in drug shop clients and vendors. Please mention it. | The sample of clients and vendors were evenly split between those from drug shops in major trading centers vs. more remote villages, and this detail has been added. |
| Methods | In the sample size calculation, details of the total number of VHTs and targeted health officials in the communities are not mentioned. | The total number of VHTs in Bugoye has been added. Because health and community officials were individually selected based on study team discussions, no sampling frame was created. |
| Methods | Page Number 7, table 1, citation of the previous study is missing on whose data, RDT sales cutoff developed. | Citation has been added. |
| Methods | The tabulation form of the explored theme of all participant's categories can reflect a better understanding of the individual participant's category interview structuring. | Table 2, which outlines themes covered in qualitative interviews, by participant category, has been added to the paper. |
| Methods | Please mention the interview guide details their level of expertise and if they were trained for the interview before the data collection. | Information about interviewers was added, specifically that they were “from the region, had experience conducting research with low-literate participants, and received training on study procedures, qualitative research best practices, and research ethics.” |
| Methods | A table with socio-demographic details (age, sex, education, residency status, family income) and health and community officials' categories of the participants can provide additional insights. | No socio-demographic details were collected from interview participants and therefore cannot be included in this paper. Any further information about the positions of health and community officials has been deliberately excluded to avoid the possibility of deidentification. |
| Ethical Considerations | The study mentioned monetary incentives to each health and community official. Please explain the reason and category of incentive. It was provided against the wage loss, travel, or any other allowance. | It has been added that the incentives were provided as a token of appreciation for their participation in the study. |
| Results | Findings can also be mentioned in major subtheme, apart from identified themes, though it's mentioned in supplementary data. | We chose to describe the findings by identified themes to allow for better integration of results across all types of interviewed participants. |
| Results | Page Number 9, line 211, you mentioned "initial point of care" please mention if its febrile status or preventive diagnosis. The study excludes febrile patients and severely affected patients with limited or no movement due to the disease. | It has been clarified that this was for fever specifically. The study did not exclude febrile patients or severely affected patients – several interviewed clients were seeking treatment for an individual sick at home. |
| Results | page number 10, line number 247, In the themes of perception of rapid diagnosis test VHTs members perception can be added in one or two statements to make it wider perspective.' | VHTs and drug shop vendors had similar perceptions of RDTs, with overwhelmingly favorable opinions and the same reasons for liking RDTs. This section has been revised to reflect that the findings described apply to both VHTs and drug shop vendors. |
| Results | Page number 10, line 263-264, mention the % along with the number for better clarity. | As a qualitative paper, we have tried to avoid quantifying our results and have relied on qualifiers whenever possible (i.e., most, some). In some instances, such as this one, we have included numbers to address stark differences. Including a single percentage would further quantify the results in a way that is inconsistent with the rest of the paper. |
| Results | Page number 11, line number 290, In Theme use of RDT you mentioned "Among the twelve clients who purchased RDTs", the number should be 13, as mentioned in table 1. | Thank you for catching this! That has been corrected. |
| Results | Page number 13, line number 323, you highlighted the importance of income seasonality and malaria testing preference; please discuss in the discussion part whether the income and high transmission season overlap in the community. | Additional detail on when the periods of high income occur has been added to the discussion. |
| Results | Page number 13, line number 346, information about antipyretic medication can be discussed if explored whether clients are considering the antimalarial as antipyretic. | Unfortunately, we don’t have very detailed information about how clients viewed the function of antimalarials that can be added to the manuscript. |
| Discussion | Page number 16, line number 414. In the Theme intervention recommendation author mentioned "how to include vendors who don't meet the necessary qualification" please discuss in the light of previous studies if any KAP survey was done. Please incorporate it in the discussion part. | No formal knowledge, attitudes, and practices survey was conducted among drug shop vendors. Qualifications here refers specifically to educational requirements. This has been clarified in the text. |
| Discussion | Strengths and weaknesses of the study may be cited in the manuscript.  The one limitation of this work is the small sample size while clustering the data. More in-depth research is needed based on the study observation.  The study aims to capture the community perceptions about RDTs and to identify the barriers to use of RDTs. Hence, in-depth interviews should have been conducted with those community members as well who did not visit the drug shops. Not including these members of the community may induce bias in the study as their characteristics and reasons thereof for not using RDTs may be systematically different from clients coming to the drug shops for RDTs. | A strengths and limitations section has been added to the discussion. |
| Conclusion | On page number: 21 and line number 557, the author mentioned, "This study provides in-depth information on the root causes of overtreatment for malaria at drug shops in Bugoye". However, seems does not seem to match with the study's main findings; based on the two direct responses of the drug clients on their previous episode, it may not be drawn, rather should be "underuse of RDT". Please review the statement and reframe it. | This statement has been reframed to more closely reflect study findings and conclusions, by specifying that it “explored reasons for the low use of RDTs and nonadherence to negative RDT results.” |
| References | Please review your reference list to ensure that it is complete and correct. If you have cited papers that have been retracted, please include the rationale for doing so in the manuscript text, or remove these references and replace them with relevant current references. Any changes to the reference list should be mentioned in the rebuttal letter that accompanies your revised manuscript. If you need to cite a retracted article, indicate the article’s retracted status in the References list and also include a citation and full reference for the retraction notice. | We have reviewed the reference list for completeness and have ensured no retracted articles have been included. |
| References | Some of the references are not directly related to the cited details and make the lengthy list Please refer to page number 3, line number 72. (5,7, 9-14), if the reference is not cited in other study details, omit them. | References not cited in other study details omitted from reference list. |
| References | References poorly compiled and not in accordance with either the Vancouver / ICMJE style. Page numbers missing in several of the references cited. Suggestion: Follow one particular reference style, either Vancouver or ICMJE style as per the journal’s requirement.  Some of the references are not in the prescribed format, please check it once again to make it uniform and systematic. Please see: Reference number: 7, 8. 10, 15, 35, 45 Some references are referring the supplementary data. Please check 19. | The reference list has been revised in accordance with the Vancouver style using Mendeley reference manager software to ensure uniformity. Specific cited references have been revised. |
